# Beyond cell-cell adhesion: Plakoglobin and the regulation of tumorigenesis and metastasis

**DOI:** 10.18632/oncotarget.15650

**Published:** 2017-02-23

**Authors:** Zackie Aktary, Mahsa Alaee, Manijeh Pasdar

**Affiliations:** ^1^ Department of Oncology, University of Alberta, Edmonton, Alberta, Canada; ^2^ Institut Curie, Orsay, France

**Keywords:** Plakoglobin, γ-catenin, tumor/metastasis suppressor, p53, gene expression

## Abstract

Plakoglobin (also known as? -catenin) is a member of the Armadillo family of proteins and a paralog of β -catenin. Plakoglobin is a component of both the adherens junctions and desmosomes, and therefore plays a vital role in the regulation of cell-cell adhesion. Similar to β -catenin, plakoglobin is capable of participating in cell signaling in addition to its role in cell-cell adhesion. In this context, β -catenin has a well-documented oncogenic potential as a component of the Wnt signaling pathway. In contrast, while some studies have suggested a tumor promoting activity of plakoglobin in a cell/malignancy specific context, it generally acts as a tumor/metastasis suppressor. How plakoglobin acts as a growth/metastasis inhibitory protein has remained, until recently, unclear. Recent evidence suggests that plakoglobin may suppress tumorigenesis and metastasis by multiple mechanisms, including the suppression of oncogenic signaling, interactions with various proteins involved in tumorigenesis and metastasis, and the regulation of the expression of genes involved in these processes. This review is primarily focused on various mechanisms by which plakoglobin may inhibit tumorigenesis and metastasis.

## INTRODUCTION

**Figure 1:**
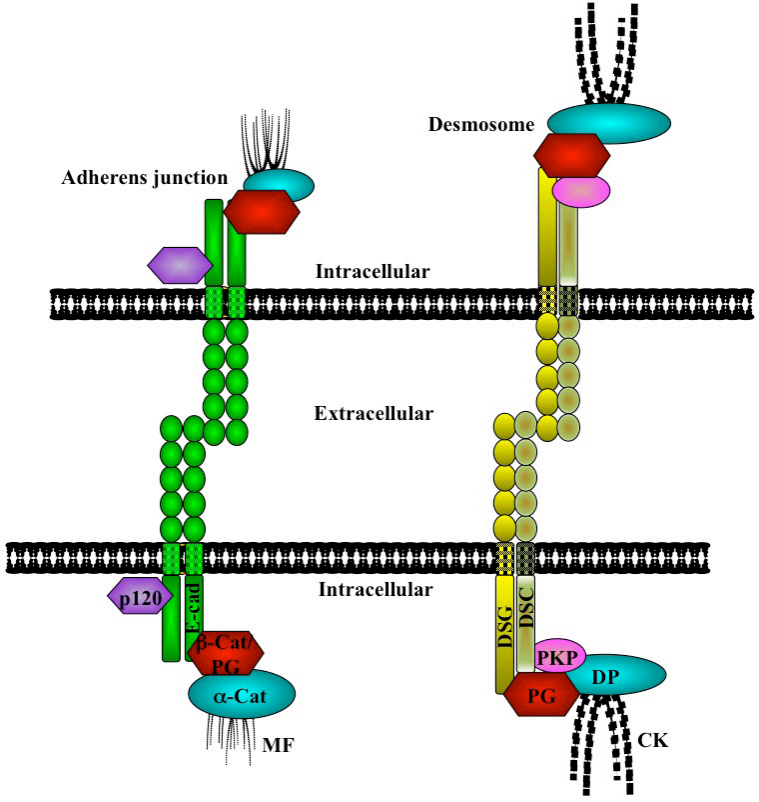
Cell adhesion complexes in epithelial cells Cell-cell adhesion is maintained in epithelial tissues by the adherens junction and desmosomes. At the adherens junctions, E-cadherin (E-cad) forms extracellular interactions with E-cadherin molecules on neighboring cells. Intracellularly, E-cadherin interacts with either β-catenin (β-cat) or plakoglobin (PG), which then interact with α-catenin (a-cat), an actin binding protein. A fourth catenin, p120-catenin, also interacts with E-cadherin and regulates its stability at the membrane. The E-cadherin/catenin complex is stabilized at the membrane by interaction with the actin microfilaments (MF). At the desmosome, the desmosomal cadherins [desmoglein (DSG) and desmocollin (DSC)] interact with plakoglobin (PG) and plakophilin (PKP), which interact with desmoplakin (DP), which in turn associates with the cytokeratin intermediate filaments (CK). The basic, core protein composition of the desmosomes is represented here: the exact protein constituents of the desmosomes and their interactions vary among different types of cells and tissues. (See the text for references).

Epithelial tissues cover the surface of the body and line the internal cavities [1]. The structural integrity of these tissues requires extensive cell-cell adhesion and interactions mediated by the adhesive junctional complexes consisting of the adherens junctions and desmosomes [2–5]. Adherens junctions are a ubiquitous type of intercellular junction and are present in both epithelial and non-epithelial cells [3, 6], whereas desmosomes are adhesive junctions that confer tensile strength and resilience on cells and are present not only in epithelial cells but also in non-epithelial cells that endure mechanical stress, such as cardiac muscle and meninges [7. 8]. Both adherens junctions and desmosomes are cadherin based. Cadherins are single-pass transmembrane glycoproteins that form homotypic interactions with cadherin proteins on neighboring cells and interact intracellularly with proteins of the catenin family [4, 5]. At the adherens junction in epithelia, the C-terminal domain of E-cadherin interacts, in a mutually exclusive manner, with β-catenin or γ-catenin (plakoglobin), which then interacts with α-catenin, an actin-binding protein. A fourth catenin protein, p120-catenin, interacts with the juxtamembrane domain of E-cadherin and stabilizes the cadherin dimers at the membrane (Figure 1; [9,10]). At the desmosome, the cytoplasmic domain of the desmosomal cadherins (desmocollins and desmogleins) interacts with plakophilin and plakoglobin, which in turn are associated with desmoplakin, an intermediate filament binding protein that connects the complex to the cytoskeleton (Figure 1; [7, 11]).

Although originally identified as structural proteins with a “glue-like” function, cadherin-based cell adhesion complexes (adherens junctions and desmosomes) have subsequently been shown to have significant interactions with elements of the signal transduction pathways that regulate growth and morphogenesis [12–17]. More specifically, cadherin-independent β-catenin and plakoglobin have been shown to have signaling functions through their interactions with an array of functionally diverse proteins, including receptor tyrosine kinases and phosphatases, tumor suppressors and transcription factors [18–20]. Consequently, these catenin proteins play important roles in regulating tumor development and metastasis progression. β-catenin has a well-documented oncogenic potential as a component of the Wnt signaling pathway, whereas plakoglobin generally acts as a tumor/metastasis suppressor. While some studies have reported a tumor promoting activity of plakoglobin in a cell/malignancy specific context as discussed in the following sections, this review is primarily focused on the potential mechanisms by which plakoglobin may inhibit tumorigenesis and metastasis.

## PLAKOGLOBIN: INITIAL IDENTIFICATION AND EARLY CHARACTERIZATION

Plakoglobin was originally identified as an 83 kDa protein component of the desmosomal plaque [21). Following its initial identification, experiments using monoclonal antibodies, cDNA cloning and a combination of biochemical, morphological and molecular approaches demonstrated that this 83 kDa protein was present in both desmosomes and adherens junctions and thus, was given the name plakoglobin [21, 22).

Although identified as a junction protein, the role that plakoglobin played in the junctional complexes was unclear, and the partners it interacted with remained unidentified. Several years later, coimmunoprecipitation experiments showed that plakoglobin interacted with both desmoglein (thereby confirming plakoglobin as a constituent of the desmosomes; [23] and E-cadherin [24, 25]. However, since plakoglobin was found to be only loosely associated with E-cadherin, it was suggested that the main adhesive complexes primarily consisted of E-cadherin, β-catenin and α-catenin, although the existence of a separate E-cadherin-plakoglobin-α-catenin complex could not be ruled out [25].

Subsequently, it was demonstrated that plakoglobin interacted with both desmoglein and E-cadherin in both the soluble and cytoskeleton-associated pools of cellular proteins. Furthermore, a distinct, cadherin-independent pool of plakoglobin was observed, which suggested that plakoglobin may have a cellular function independent of cell adhesion [26]. Finally, phosphorylation experiments revealed that whereas the insoluble (cadherin-associated) pool of plakoglobin was serine phosphorylated, the soluble pool was serine, threonine and tyrosine phosphorylated, suggesting that these different pools of plakoglobin are differentially regulated and may potentially perform varying functions [26].

## PLAKOGLOBIN AND CELL-CELL ADHESION

The most documented role of plakoglobin within the cell is in cell-cell adhesion. The identification of plakoglobin as a component of both the adherens junction and desmosomes suggested that it plays an important role in regulating cell-cell adhesion. However, since it was observed that the adherens junction could exist as a complex containing E-cadherin, β-catenin and α-catenin, independent of plakoglobin [25], the requirement for plakoglobin at the adherens junctions remained in question. Regardless, the essential role of plakoglobin in the regulation of cell-cell adhesion soon became apparent.

The function of plakoglobin in regulating cell-cell adhesion was clearly demonstrated when it was shown that the re-expression of E- or P-cadherin in cadherin-null murine spindle cell carcinomas with very low levels of plakoglobin was not sufficient to modify the morphology or tumorigenicity of the cells [27]. In these cells, although the exogenously expressed cadherins localized to the cell membrane and interacted with both α- and β-catenin, they did not interact with plakoglobin and desmosomes were not formed. From this work, the authors concluded that the presence of plakoglobin in the E-cadherin complex may be necessary for proper cell-to-cell adhesion.

The role of plakoglobin in regulating junction formation was also demonstrated when it was shown that A431 epithelial cells treated with dexamethasone (which decreased E-cadherin and plakoglobin levels) were unable to form adherens junctions and desmosomes and exhibited a fibroblastic morphology. Following the expression of E-cadherin in these cells, the adherens junctions were formed but the fibroblastic morphology of the cells remained unchanged. The authors then expressed an E-cadherin-plakoglobin chimeric protein in these cells, which resulted in the formation of stable adherens junctions and desmosomes and induced an epithelioid morphology. Together, these results suggested that E-cadherin-plakoglobin interactions were necessary for the formation of stable adhesive complexes and provided the first indication that plakoglobin served as a molecule involved in the cross-talk between the adherens junctions and desmosomes [28].

Following this study, our laboratory demonstrated the role of plakoglobin in junction formation by expressing plakoglobin in SCC9 cells, a human tongue squamous cell carcinoma cell line that lacks the expression of both plakoglobin and E-cadherin but expresses N-cadherin [29, 30]. Transfectants expressing E-cadherin (SCC9-E) or low/physiological levels of plakoglobin (SCC9-PG) or both, were generated and showed that the independent expression of either E-cadherin or plakoglobin induced a mesenchymal (transformed) to epidermoid (normal) phenotypic transition (MET). This phenotypic transition was associated with decreased cell proliferation and increased cell-cell adhesion, with only SCC9-PG cells capable of forming desmosomes. E-cadherin or plakoglobin expression also coincided with decreased soluble β-catenin levels, while E-cadherin expression downregulated N-cadherin and, plakoglobin expression increased N-cadherin levels and stability [29, 30]. Since then, numerous subsequent studies identified the switch from E- to N-cadherin as a major contributing factor in the epithelial to mesenchymal phenotypic transition (EMT) and metastatic progression. Significantly, our results clearly demonstrated that in the absence of E-cadherin, plakoglobin was able to inhibit N-cadherin tumor promoting activities and that the cadherin switch by itself cannot explain the transformed phenotype of SCC9 cells. Furthermore, the induction of MET by E-cadherin and plakoglobin may occur via a common pathway that also involves β-catenin [29, 30].

Other studies have characterized further the role of plakoglobin in desmosome assembly and function, demonstrating that plakoglobin is essential for the proper assembly of the desmosomal plaque and the efficient binding of desmoplakins to the intermediate filaments [31, 32].

## PLAKOGLOBIN AND EMBRYONIC DEVELOPMENT

While the above studies were aimed at understanding the role of plakoglobin in the formation and regulation of adhesive junctional complexes in tissue culture cells, plakoglobin knockout transgenic mouse models were also generated to study the role of plakoglobin during embryonic development. Work from two independent laboratories demonstrated that the homozygous knockout (double knockout) of plakoglobin resulted in embryonic lethality in mice [33, 34]. Analysis of mouse embryos from different stages of embryogenesis revealed that plakoglobin double knockout embryos were phenotypically similar to wild type and heterozygous embryos until approximately E8.5 and E9.5 [33]. In E10.5 and E12.5 embryos, it was observed that plakoglobin double knockout mice displayed severe heart defects (e.g. thin and weak heart walls). There were considerably fewer desmosomes in these embryos and their plaques were less dense, particularly in the intercalated discs of cardiac muscle [33, 34]. Therefore, these studies demonstrated that the loss of plakoglobin during embryonic development resulted in lethality, which could be attributed to defective desmosome formation and impaired cardiac development.

Similar to plakoglobin, the loss of β-catenin during embryonic development also resulted in lethality. However, β-catenin double knockout mouse embryos began to display defects earlier than plakoglobin double knockout embryos. At E7.0, β-catenin double knockout embryos showed defects in the embryonic ectodermal layer, in which cells became detached from the layer and were dispersed in the proamniotic cavity [35]. Furthermore, at E7.5, β-catenin double knockout embryos were half as large as wild type and heterozygous embryos and histological analyses demonstrated that the three germ cell layers had not formed. Most notably, the normal epithelial organization of the ectodermal layer was completely absent [35]. Together, these studies showed distinct roles for plakoglobin and β-catenin in embryonic development; whereas knockdown of plakoglobin resulted in defects in cardiac structure, β-catenin knockdown resulted in impaired migration of ectodermal cells.

## PLAKOGLOBIN AND CELL SIGNALING

### i. Overview

**Figure 2:**
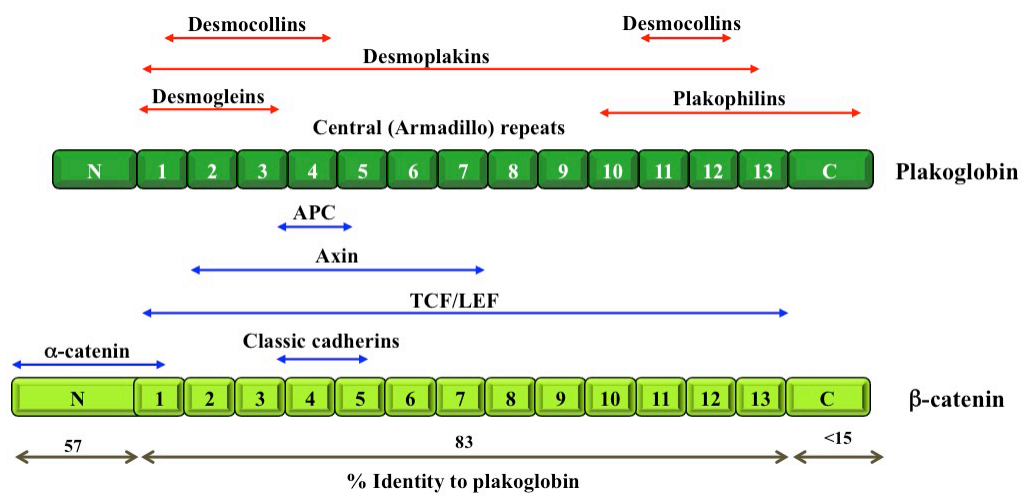
Schematic structure of β-catenin and plakoglobin Both β-catenin and plakoglobin contain 13 Armadillo repeats that are flanked by N- and C-terminal domains, respectively. The degree of identity between β-catenin and plakoglobin for each domain is indicated. Blue arrows indicate protein partners that interact with both β-catenin and plakoglobin and the domains involved in these interactions. Red arrows indicate proteins that interact with plakoglobin only and the domains involved in these interactions. APC, adenomatous polyposis coli; TCF/LEF, T-cell factor/Lymphoid enhancer factor (see Table 1 for references).

As mentioned above, in addition to their roles in regulating cell-cell adhesion, catenin proteins participate in cell signaling through their interactions with various intracellular partners. Catenin-mediated cell signaling has been the focus of many studies, most of which have concentrated on β-catenin and overlooked plakoglobin. Plakoglobin and β-catenin are paralogs and members of the Armadillo family of proteins (Figure 2; [36]). As such, they share common intracellular partners, including classical cadherins, α-catenin, axin, APC and TCF/LEF [18, 37–41]. Despite their structural similarities and their common interacting partners, plakoglobin and β-catenin appear to play opposite roles with respect to cell signaling in tumorigenesis and metastasis. β-catenin has a well-defined oncogenic potential as a component of the Wnt signaling pathway [18, 19, 42, 43], whereas plakoglobin has been typically associated with tumor and metastasis suppressor activities through mechanisms that are beginning to be deciphered [29, 44–58]. In the following sections, we describe experimental evidence, which links plakoglobin to various signaling pathways that regulate tumorigenesis and metastasis.

### ii. Wnt signaling pathway

The Wnt pathway is a signaling cascade with fundamental roles in the regulation of cell proliferation, cell polarity and cell fate determination during embryonic development and in tissue homeostasis. Deregulation of this pathway results in birth defects and various diseases, including cancer (for reviews, see [19, 43, 59]).

The first indication that plakoglobin might participate in the Wnt pathway (and in turn, cell signaling) was when its levels were increased in PC12 cells following exogenous Wnt-1 expression. In addition to its increased levels, plakoglobin underwent membrane redistribution, suggesting that in addition to β-catenin levels, Wnt-1 can modulate plakoglobin levels and localization [60]. Subsequently, it was demonstrated that the microinjection of mRNAs encoding plakoglobin into fertilized *Xenopus* embryos resulted in dorsalized gastrulation and anterior axis duplication [61]. In this study, the exogenously expressed plakoglobin localized at both the plasma membrane and in punctate nuclear aggregates. Importantly, when mRNAs encoding plakoglobin and the cytoplasmic domain of desmoglein were co-injected into the embryos, both dorsalized gastrulation and anterior axis duplication were suppressed. In these embryos, plakoglobin was localized primarily to the plasma membrane with some peri-nuclear distribution, suggesting that junction-independent plakoglobin has signaling ability similar to β-catenin.

While this initial study suggested that plakoglobin may have signaling functions similar to β-catenin, several lines of evidence suggest that this is most likely not the case.

The Klymkowsky group has shown that membrane-anchored forms of plakoglobin produced the same axis duplication as the wild type protein [62]. demonstrating that nuclear plakoglobin was inconsequential in inducing a Wnt-like phenotype. The observation that plakoglobin was ineffective in Wnt signaling was also made in *Drosophila*, when transgenes encoding either β-catenin or plakoglobin were expressed in embryos which lacked functional armadillo protein. Using the ectodermal expression of *engrailed* as an indicator of Wnt signaling, the authors observed that while zygotic expression of β-catenin resulted in weak *engrailed* expression, plakoglobin was unable to induce the same the phenotype [63].

Several other groups have shown that compared to β-catenin, plakoglobin has limited signaling activity in the context of the Wnt pathway. Simcha *et al*. [64] showed that LEF-1 overexpression in MDCK cells resulted in the nuclear translocation of β-catenin but not plakoglobin. In the same study, TOPFLASH reporter activity was considerably higher in HEK293T cells transfected with β-catenin compared to those transfected with plakoglobin expression constructs [64]. Furthermore, expression of a transcriptionally active β-catenin mutant, S37A, induced TOPFLASH reporter activity in 293T cells to a much greater extent than that by the analogous S28A plakoglobin mutant [41]. Consistent with these results, electrophoretic mobility shift assays using *in vitro* translated β-catenin, plakoglobin, TCF-4 and LEF-1 and radioactively labeled DNA corresponding to TCF/LEF binding sequences showed that β-catenin-TCF-4/LEF-1-DNA complexes were efficiently formed, whereas plakoglobin-TCF-4/LEF-1-DNA complexes were not detected [65, 66]. Taken together, these results suggest that while plakoglobin may have potential signaling activity as part of the Wnt pathway, this activity is minimal, especially when compared to that of β-catenin.

It is important to mention that a small number of *in vitro* studies have shown that plakoglobin expression resulted in increased cell proliferation, migration and invasion [67, 68], which would be consistent with an oncogenic signaling activity of plakoglobin. It must be noted, however, that in these few cases, plakoglobin was overexpressed in cells that already contained high levels of endogenous β-catenin [69, 70]. Previous work by several groups, including ours, has shown that overexpressed plakoglobin promotes the oncogenic signaling activity of β-catenin by interacting with proteins that normally sequester β-catenin away from the nucleus [64, 71–74]. Therefore, in the few studies that determined that plakoglobin has oncogenic signaling activity, the overexpressed plakoglobin most likely sequestered β-catenin interacting partners, allowing for the liberation of β-catenin and activation of its oncogenic activity (see [18] and the following section on “Inhibition of β-catenin oncogenic signaling”).

### iii. Sonic hedgehog signaling pathway

The sonic hedgehog pathway plays an important role in the proper development and patterning of the limbs, brain, musculature, skeleton and lungs, as well as in the renewal of adult stem cells and tumorigenesis [75, 76]. Gli1, a transcription factor that is activated following stimulation of the sonic hedgehog pathway, has been shown to activate the expression of plakoglobin in human rhabdomyosarcoma cells by binding to a Gli1 responsive element in the human plakoglobin gene (*JUP*) promoter [77]. In addition, Gli1 promotes plakoglobin expression in medulloblastoma, whereas it represses plakoglobin in glioblastoma [78, 79]. These results suggest that plakoglobin, as a target of Gli1 and the sonic hedgehog pathway, may be involved in different signaling pathways that regulate tumorigenesis and metastasis. However, it is not clear what role (if any) plakoglobin plays in the sonic hedgehog pathway.

### iv. Src signaling

Experimental evidence from different groups has suggested that there is an inverse relationship between Src signaling and plakoglobin. Specifically, plakoglobin may inhibit cell migration through the regulation of Src signaling [55] and Src kinase may downregulate plakoglobin expression and/or phosphorylation, inhibiting plakoglobin's tumor suppressor activities, thereby promoting migration [80–82]. Plakoglobin was recently shown to be present in c-Src containing lipid rafts [83] in association with the raft membrane proteins flotillins [84], which are also phosphorylated by c-Src [85]. Prior work on the role of plakoglobin in migration and invasion showed that when MCF-7 cells were treated with human growth hormone (hGH), plakoglobin levels were decreased and cell migration and invasion were increased. Interestingly, this hGH-mediated invasive phenotype was dependent on Src signaling, since chemical inhibitors of Src resulted in increased plakoglobin levels and in turn, decreased invasion and migration [86]. These observations were further confirmed by Shafiei *et al*. [82], who demonstrated that hGH downregulated plakoglobin expression via the activation of Src and JAK2 kinases. The authors also showed that these kinases stimulated the mRNA and protein expression of DNA methyltransferase 1 (DNMT1), DNMT3A and DNMT3B, which in turn led to the hypermethylation of the plakoglobin (*JUP*) promoter and its decreased expression [82]. Consistent with these *in vitro* studies, the GH receptor was shown to be overexpressed in both epithelial and stromal components of axillary lymph node metastasis in breast tumors. This overexpression of the receptor was also associated with decreased plakoglobin expression in nodal metastasis [87]. Another recent study using 28 non-small cell lung cancer (NSCLC) cell lines, and a combination of *in vitro* and *in vivo* mouse xenograft experiments showed a significant reduction in tumor growth in 68% of the cell lines upon combined inhibition of the Src and MAPK pathways. The combination drug treatment was shown to induce MET concurrent with the upregulation of plakoglobin and E-cadherin and downregulation of Snail1, FAK and PAX expression [88].

Finally, plakoglobin was shown to regulate cell-extracellular matrix (ECM) adhesion and motility via ECM-dependent Src activation and inhibit the migration of single keratinocyte cells by regulating the deposition of fibronectin and vitronectin, organization of the actin cytoskeleton and RhoGTPases [49, 51, 55].

### v. Ras signaling

The phosphorylation of plakoglobin by a Ras-dependent pathway was initially reported by Hegland *et al*. [89], who showed that the expression of a dominant negative Ras (N17Ras) inhibited the increased expression of plakoglobin in confluent cultures of endothelial cells and inhibited the formation of 3-dimensional vascular structures [89]. These observations were later supported by increased levels of cadherin/catenins (α, β, γ/plakoglobin) in cultures of breast, colon and liver cancer cells treated with FTI-277, which inhibits Ras farnesylation and disrupts MAPK activation [90, 91]. Treatment with FTI-277 also led to a significant reduction in *in vivo* tumor growth and metastasis in SCID mice, relative to the control animals, when treated for 3 weeks following cancer cell inoculation [91]. Finally, a recent report has suggested that plakoglobin can suppress the oncogenic signaling activity of K-Ras. In this study, the expression of the oncogenic K-Ras (K-Ras12V) in Rat2 cells led to the decreased plakoglobin levels. Furthermore, decreased plakoglobin levels were accompanied by decreased levels of the histone deacetylase HDAC4, and increased cell migration and invasion. The subsequent exogenous expression of plakoglobin, but not β-catenin, in the Rat2-K-Ras12V expressing cells resulted in increased HDAC4 protein and decreased migration and invasion. The increased HDAC4 following plakoglobin expression was dependent on LEF-1, since LEF-1 knockdown in the plakoglobin expressing Rat2-K-Ras12V cells resulted in loss of HDAC4 expression [92]. At this point, however, it is not clear whether plakoglobin itself regulates the expression of HDAC4.

## ROLE OF PLAKOGLOBIN IN STEM CELLS, CANCER STEM CELLS AND CIRCULATING TUMOR CELLS

### i. Plakoglobin and stem/cancer stem cells

**Figure 3:**
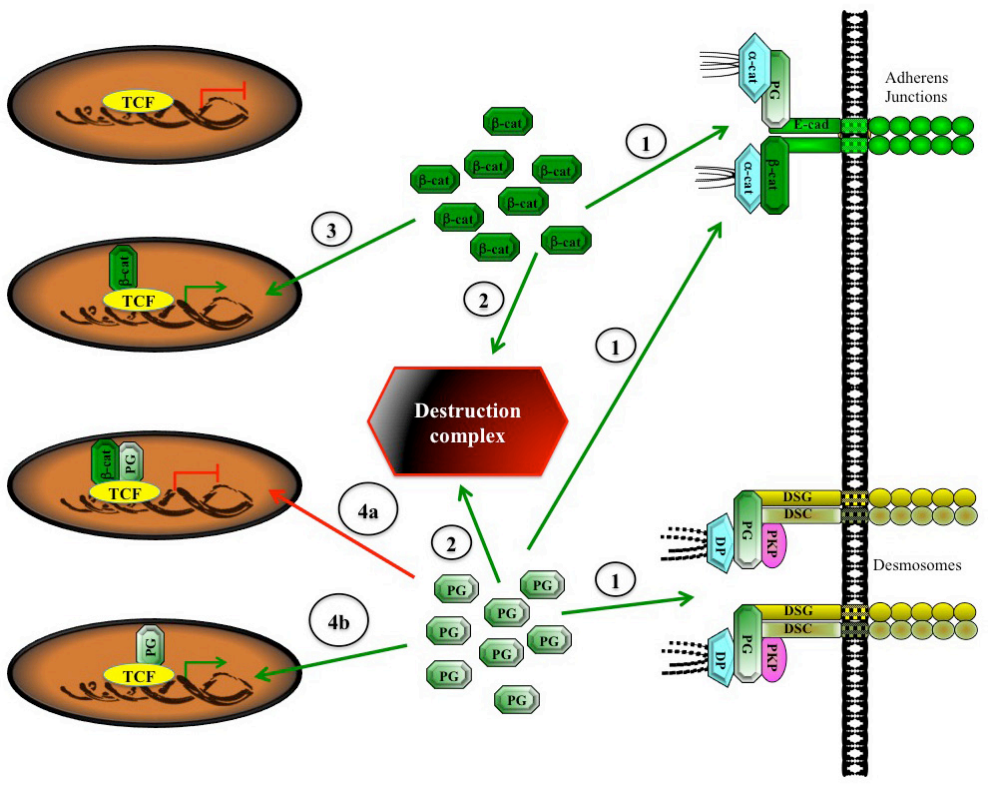
A Potential model of indirect and direct activation of TCF transcriptional activity by plakoglobin Upon synthesis, β-catenin interacts with cadherins at the adherens junctions, whereas plakoglobin interacts with cadherins at the adherens junctions and desmosomes (1). The excess β-catenin and plakoglobin proteins are recruited to the destruction complex and degraded via the proteasome pathway (2). Excess cytoplasmic β-catenin can translocate to the nucleus, bind to TCF and change its activity from a repressor to an activator (3). The excess cytoplasmic plakoglobin can take one of several fates, depending on the level of β-catenin and its nuclear presence. If the excess plakoglobin remains in the cytoplasm, it can indirectly activate TCF by replacing β-catenin in adherens junctions, allowing for the interaction of the liberated β-catenin with TCF and its transcriptional activation (3). Alternatively, if the excess plakoglobin translocates to the nucleus, it can suppress β-catenin-TCF activation (4a), or in the absence of nuclear β-catenin, it can interact with TCF and directly lead to its transcriptional activation (4b). α-cat, α-catenin; β-cat, β-catenin; E-cad, E-cadherin; PG, plakoglobin, DSG, desmoglein, DSC, desmocollin; PKP, plakophillin, DP, desmoplakin; TCF, T-cell factor.

A recent study has assessed the role of plakoglobin in the differentiation of mouse embryonic stem cells (mESCs) in the context of the Wnt pathway [93], which is essential for ESC pluripotency [94, 95]. This study compared Wnt stimulated β-catenin^+/+^ and β-catenin^−/−^ mESCs. While Wnt stimulation in β-catenin^−/−^ mESCs increased plakoglobin levels, the suppression of plakoglobin had no effect on Wnt targets. In contrast, ectopic overexpression of plakoglobin in wild type mESCs, strongly activated Wnt target genes, stabilized the expression of pluripotency markers Sox2, Oct4 and Nanog and inhibited mESCs differentiation. These effects were partially due to the stabilization of the transcriptionally active β-catenin owing to the overexpression of plakoglobin (also see below and Figure 3). Interestingly, it was recently shown that disabled-2 (DAB-2), a negative regulator of Wnt signaling and embryonic development, interacts with plakoglobin. It was further demonstrated that DAB-2 knockdown in mESCs had no effect on pluripotency but inhibited differentiation [96–98]. Together, these results suggest that while βcatenin is involved in the maintenance of mESC pluripotency, plakoglobin may promote mESC differentiation.

Several studies have suggested a role for plakoglobin in the maintenance of stem cell properties in the context of the Wnt/β-catenin signaling pathway and its role in hematopoiesis and hematopoietic malignancies [99, 100]. Interestingly, earlier studies showed that normal multilineage hematopoiesis was independent of β-catenin and plakoglobin [101, 102]. In contrast, subsequent numerous studies have shown the involvement of the canonical Wnt/β-catenin pathway in the regulation of hematopoietic stem cell (HSC) properties, specifically self-renewal vs. differentiation [reviewed in 103]. Both β-catenin and plakoglobin are expressed during normal hematopoietic development and in HSCs and HSPCs (hematopoietic stem progenitor cells) [73]. The same study showed that plakoglobin knockdown induced a decrease in M-CSFR (macrophage colony-stimulating factor receptor, CD115) levels and interfered with monocyte lineage differentiation. Furthermore, overexpression and nuclear localization of plakoglobin increased the expression of the transcription factor PU.1, a master regulator of myeloid differentiation and maintenance of monocyte lineage commitment. The authors also demonstrated that nuclear plakoglobin upregulated PU.1 expression via relieving PU.1 repression by β-catenin/TCF [73].

Among hematopoietic malignancies, the abnormal expression/activity of β-catenin has been well documented in leukemia (reviewed in [104]). Although increased expression of plakoglobin has also been reported, it is not clear whether plakoglobin is involved directly in leukemogenesis or indirectly via the activation of β-catenin. In acute myeloid leukemia (AML), AML-associated translocation products (AATPs) activated the Wnt signaling pathway and self-renewal of HSCs. This Wnt activation was concurrent with the upregulation of plakoglobin expression, increased β-catenin protein levels and activation of β-catenin-LEF target genes including c-Myc and cyclin D1 [105–108]. However, none of these studies examined the role of plakoglobin in the self-renewal of HSCs in the absence of β-catenin. It is well known that increased plakoglobin levels can activate β-catenin signaling in epithelial cells [71, 72]. In the case of leukemia, Niu *et al*. [109] also showed that knockdown of BCR-ABL in chronic myeloid leukemia (CML) cells downregulated plakoglobin expression and suppressed β-catenin by activating GSK-3β [109]. The same group also reported that Wnt5a enhanced the inhibitory effect of imatinib mesylate on CML cell growth by decreasing the levels of plakoglobin concurrent with increasing JNK and suppressing β-catenin signaling activities, respectively [74]. Yet another study by Morgan *et al*. [73] reported that in normal CD34^+^ HSPCs cells, both β-catenin and plakoglobin were expressed and distributed exclusively in the cytoplasm. In contrast, in AML patients and cell lines, plakoglobin was frequently overexpressed and its overexpression was concurrent with β-catenin stabilization and nuclear localization. This study also showed TCF-dependent transcriptional activation of plakoglobin in β-catenin knockdown cells. The authors concluded that the aberrant activation of the Wnt pathway in AML resulted from the overexpression of plakoglobin, stabilization of β-catenin, abnormal nuclear localization of both proteins and activation of the TCF-dependent transcription. The ability of plakoglobin to activate TCF-dependent transcription in the absence of β-catenin was also demonstrated by analysis of the expression of β-catenin and plakoglobin in the chronic phase (CP) and blast crisis (BC) of patients with CML. This study showed a significant negative correlation between β-catenin and plakoglobin in AP/BC cases [73]. The increased expression of plakoglobin in BC cases was concurrent with increased expression of survivin, a β-catenin-TCF-CBP target gene. Furthermore, ICG-001, a specific small molecule inhibitor of CBP, which is currently in early phase of clinical trials for various solid tumors and leukemias, was able to inhibit both β-catenin- and plakoglobin-TCF-CBP activation and survivin expression [110–113].

Together, these studies suggest that, in general, β-catenin is transcriptionally activated in leukemia. However, plakoglobin overexpression may also contribute to hematological malignancies indirectly, by promoting the nuclear localization of β-catenin and activation of β-catenin-TCF transcriptional activity. Furthermore, in the absence of β-catenin, plakoglobin can interact with TCF, activate TCF-dependent transactivation and contribute to the development of leukemia. These possibilities are supported by similar observations in epithelial cells *in vitro* [71,72]. However, unlike hematopoietic cells that lack junctional complexes, the overexpression of plakoglobin in epithelial cells *in vivo* has seldom, if ever, been reported. This is due to the sequestration of plakoglobin in adherens junctions and desmosomes via its interaction with both classic and desmosomal cadherins. In contrast, β-catenin only interacts with classic cadherins at the adherens junction and its excess (cadherin-independent pool) has to be strictly regulated by the components of the Wnt pathway. In this pathway, adenomatous polyposis coli (APC), axin, casein kinase 1 (CK1) and glycogen synthase kinase-3β (GSK-3β) form the destruction complex where the phosphorylation of β-catenin by GSK-3β and CK1 marks it for ubiquitination and degradation by the proteasome pathway. Increased β-catenin level is often observed in various carcinomas due to mutations in β-catenin itself or in the components of the Wnt pathway. In contrast, it has been reported that plakoglobin levels are generally decreased in solid tumors. The overexpressed β-catenin protein interacts with TCF and activates the expression of tumorigenic and metastatic genes. Miravet *et al*. [114] have shown that both proteins can simultaneously interact with TCF and the binding of plakoglobin to TCF that is already in complex with β-catenin hampers β-catenin-TCF transcriptional activity. However, in the absence of β-catenin, plakoglobin can activate TCF-dependent transcription *in vitro*. Thus, one can envision a scenario (Figure 3) in which β-catenin acts as the primary partner of TCF and promotes TCF transcriptional activity. When present at high levels, plakoglobin can also interact with TCF. However under these conditions, if plakoglobin associates with TCF while it is complexed with β-catenin, it will inhibit β-catenin-TCF transcriptional activation. On the other hand, if plakoglobin associates with TCF independent of β-catenin, it can activate TCF transcriptional activity, albeit at lower levels.

### ii. Plakoglobin and circulating tumor cells

Circulating tumor cells (CTC) are cells that dissociate from primary tumors, enter the circulation and form the seeds for subsequent metastatic growth in distant locations [115, 116]. CTCs are present as single cells or clusters. CTCs are known to have cancer stem cell properties and their presence has been correlated with poor prognosis in various solid tumors [117–119]. The role of cell-cell adhesion proteins E-cadherin, β-catenin and plakoglobin in CTCs has been described in the context of cell-cell adhesion and its role in cell survival. Several studies have shown the overexpression of the E-cadherin/α-catenin/β-catenin complex in lymphovascular emboli (CTC clusters) of inflammatory breast cancers [120–122]. These emboli, known as lymphovascular invasion (LVI), were formed due to the decreased sialyl-Lewis X/A carbohydrate ligand-binding epitopes on MUC1 overexpressed in CTC clusters, distancing them from the endothelial cell layer. The overexpression of the E-cadherin/catenin complex was shown to be necessary for the compaction and maintenance of these clusters [120–122]. E-cadherin overexpression has also been reported in small cell lung carcinoma (SCLC) CTC cell lines and cells isolated from pleural effusion and bone metastases [123]. In breast cancer cells, using xenograft mouse models with tagged mammary tumors, Aceto *et al*. [124] showed that CTC clusters were multi-clonal collections of 20-50 tumors cells that exhibited significantly higher metastatic potential relative to single CTCs. Additionally, the clusters originated from tumors and not as a result of cell aggregation in the circulation. In this study, single cell RNA sequencing of CTCs and CTC clusters from blood samples of the same patient showed high expression of plakoglobin in the CTC clusters. In addition, the metastasis free survival was significantly lower in patients with primary tumors expressing high plakoglobin [124]. While the authors did not examine the protein levels and distribution of E-cadherin, α/β-catenin or desmosomal cadherins, they suggested that by virtue of its role both in the adherens junctions and desmosomes, high plakoglobin expression increased the stability of CTC clusters as they entered the circulation. In a subsequent report analyzing an open access database of breast cancer patients, Lu *et al*. [125] found that patients with low plakoglobin expression showed significantly better distant metastasis free and overall survival although they detected very little difference in relapse free survival between patients with high or low/medium plakoglobin expression.

In contrast to the above studies, genetic profiling of tumors from breast cancer patient with (BM^+^) or without (BM^−^) bone marrow metastasis has identified a 3-fold reduction in plakoglobin expression in primary tumors from BM^+^ relative to BM^−^ patients [126]. In another study, using MCF-7 and T47D breast carcinoma cell lines in a combination of *in vitro* and xenograft mouse models, Holen *et al*. [54] showed increased cell proliferation, migration and invasion in plakoglobin knockdown cells. They also detected a significantly high number of CTCs in the blood of mice bearing plakoglobin knockdown tumors. In yet another study, PC3 prostate cancer cells were used in an orthotopic mouse model of castration-resistant prostate cancer to generate CTC cell lines (PC3-CTC). PC3-CTCs isolated from the blood of these mice were then compared to PC3 cells with respect to their survival in suspension and their adhesive properties and resistance to anoikis. The results showed decreased E-cadherin, plakoglobin and β4-integrin and increased Bcl-2 expression in PC3-CTCs concurrent with decreased adhesion and increased resistance to anoikis [127].

Collectively, these studies suggest that decreased plakoglobin expression is associated with the shedding of tumor cells in circulation, consistent with its low expression in single CTCs. However, in CTC clusters, the upregulation of plakoglobin, E-cadherin and catenins may increase cell-cell adhesion and provide a survival advantage by stabilizing the clusters as they enter the circulation and are transported to distant locations. In support of this possibility, a recent study has shown that CD133 (prominin-1), a marker of stem cell and CSCs [128, 129], interacted with plakoglobin in ovarian clear cell carcinoma CSCs [130]. The authors showed that the knockdown of CD133 or plakoglobin led to the decreased adhesion and dissociation of CSC clusters. This study did not examine if CD133 interacted with plakoglobin directly or whether this interaction occurred at the adherens junctions or desmosomes.

## PLAKOGLOBIN TUMOR AND METASTASIS SUPPRESSOR ACTIVITY

### i. Overview

**Figure 4:**
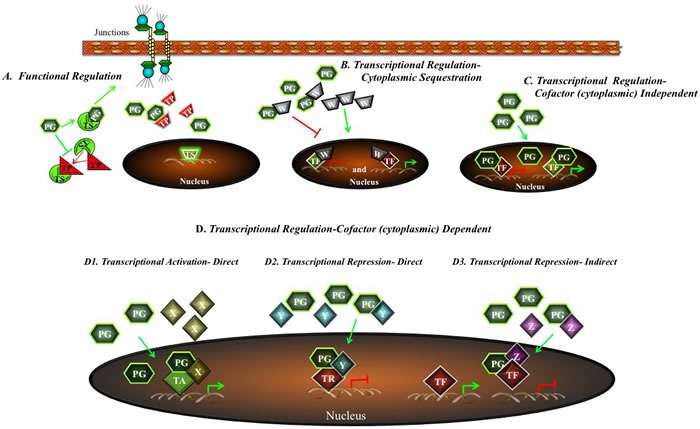
A potential model for regulation of tumorigenesis and metastasis by plakoglobin Plakoglobin may regulate tumor progression and metastasis both in the cytoplasm and in the nucleus. **A**. First, plakoglobin may interact with intracellular protein partners involved in tumorigenesis and metastasis and alter their levels, localization or function. It may also regulate gene expression via three concurrent mechanisms: **B**. Cytoplasmic Sequestration: plakoglobin sequesters a factor (W) in the cytoplasm which, in the nucleus, suppresses the expression of a tumor suppressor gene or activates the expression of an oncogene. **C**. Cytoplasmic Cofactor Independent: plakoglobin-transcription factor complexes promote the expression of tumor suppressor genes and repress the expression of oncogenes. **D**. Cytoplasmic Cofactor Dependent: plakoglobin interacts with a cytoplasmic cofactor (X, Y, Z) and this complex moves into the nucleus where it activates tumor suppressor gene expression or represses oncogenic gene expression. PG, plakoglobin; TS, tumor suppressor, TP, tumor promoter; TF, transcription factor.

Despite the observations that plakoglobin expression induces MET and the suggestion that plakoglobin suppresses tumorigenesis and metastasis, the mechanisms that could account for these activities have remained, until recently, unclear. A number of recent studies have shown plakoglobin's interaction with an array of intracellular proteins that are either directly or indirectly involved in signaling pathways that regulate tumorigenesis and metastasis (Table 1). Experimental evidence from several groups, including our own, has suggested that there are different mechanisms by which plakoglobin may act as a tumor and metastasis suppressor (Figure 4). First, plakoglobin may compete with β-catenin signaling by inhibiting TCF/β-catenin-DNA interactions and Wnt target genes expression. Second, plakoglobin may interact with various cellular partners involved in signaling and alter their levels, localization and/or functions. Finally, plakoglobin may interact with transcription factors and regulate gene expression independent of β-catenin.

**Table 1:** Plakoglobin intracellular protein partners*

Interacting partner	Function
E-/N-/VE-cadherin, PECAM	Adherens junction [158–163]**
α-catenin	Adherens junction [164]
Desmosomal cadherins, desmogleins (DSG) and desmocollins (DSC)	Desmosomes [165–167]
Desmoplakins	Desmosomes [168]
Plakophillins	Desmosomes [169–171]
CPI-17 (PKC-potentiated inhibitory protein of protein phosphatase-1)	Endothelial junction/barrier function [142]
Emmprin (Basigin, CD147)	Endothelial junctions [141]
PrP (c)-cellular prion protein	Cell-cell adhesion/barrier function and proliferation [83]
Flotillins	Cell-cell adhesion; endocytosis [84]
MUC1 (mucin1)	Adhesion, Signaling [172]
14-3-3γ	Adaptor protein involved in signaling [173]
APC	Tumor suppressor/Wnt signaling [38]
Axin	Scaffolding protein/ Wnt signaling [39]
NPM (nucleophosmin)	Nuclear proteins chaperone [56]
Nm23	Tumor/metastasis suppressor [53]
TCF/LEF	Transcription factor/Wnt signaling [114]
SOX4	Transcription factor [131]
CBP	Transcription factor [111]
p53	Transcription factor-tumor/metastasis suppressor [57]
Presenilin-1	Cell-cell adhesion, signaling [174]
CD133 (prominin-1)	Cell differentiation, proliferation, apoptosis [130]
Disabled-2 (Adapter protein that functions as a clathrin-associated sorting protein)	Cell-cell adhesion, ESC differentiation, Clathrin mediated endocytosis, signaling [98]
InsR (Insulin receptor)	Signaling [175,176]
PI3K-p85- Phosphatidylinositol 3-kinase regulatory subunit alpha	Signaling [175,176]
YAP (Yes-associated protein)	Transcriptional regulator and downstream effector of the Hippo signaling pathway [177]

*The list includes proteins that have been shown to interact with plakoglobin by co-immunoprecipitation and/or pull down assays. The cited references represent the initial report for each case only. The known functional significance of plakoglobin interaction with each partner is discussed throughout the text.

**Numbers in parentheses indicate references.

In the following sections, we will discuss the experimental evidence which suggests that plakoglobin acts as a tumor and metastasis suppressor both in the cytoplasm and nucleus, with specific emphasis on the mechanisms by which plakoglobin may accomplish such activities.

### ii. Inhibition of β-catenin oncogenic signaling

The oncogenic potential of β-catenin and the Wnt signaling pathway has been well- studied and characterized [19, 42, 59]. Following Wnt pathway activation, β-catenin translocates into the nucleus, interacts with TCF/LEF transcription factors and activates the expression of genes that promote cell survival, growth and migration (e.g. Myc, cyclin D1, MMPs, etc.; [19, 42, 59]). Interestingly, one mechanism by which plakoglobin suppresses tumorigenesis is through the inactivation of β-catenin oncogenic activity. Indeed, it was previously shown that in plakoglobin null cell lines, plakoglobin expression resulted in β-catenin displacement from the junctional complexes and its rapid proteasome-mediated degradation [71]. Similarly, we previously showed that expression of plakoglobin in SCC9 cells lacking endogenous plakoglobin expression resulted in decreased β-catenin protein levels and an induction of MET [30].

In addition to the decreasing the levels of β-catenin, plakoglobin expression can also inhibit the signaling activity of β-catenin. Comparison of the nuclear translocation and transactivation abilities of β-catenin and plakoglobin showed a highly efficient formation of β-catenin -TCF complexes, their interactions with DNA and transactivation, while plakoglobin-TCF interactions were formed inefficiently, with significantly weaker signaling activities [64]. Further analysis of the domains of the TCF/LEF transcription factors that are involved in interactions with plakoglobin and β-catenin revealed that plakoglobin and β-catenin bind to consecutive, non-overlapping regions of the TCF-4 protein. Specifically, as mentioned earlier, β-catenin bound to amino acids 1-50 in TCF-4 whereas plakoglobin bound to amino acids 51-80. Additionally, this study showed that plakoglobin can form a complex with TCF-4 and β-catenin, but that plakoglobin inhibits the ability of this complex and TCF-4 alone to interact with DNA [114]. In agreement with these findings, we previously showed that the exclusively nuclear expression of plakoglobin in SCC9 cells resulted in decreased β-catenin -TCF nuclear interactions and in turn, decreased β-catenin oncogenic signaling [72].

Plakoglobin also inhibits Wnt/β-catenin signaling through its interactions with SOX4, a transcription factor that promotes the signaling activity of β-catenin [131]. This study showed that in prostate cancer cells stimulated with Wnt3a, plakoglobin interacted with SOX4 and exported it into the cytoplasm. Furthermore, the formation of the SOX4-plakoglobin complex decreased the expression of Wnt/β-catenin target genes (e.g. Dicer, Axin2). Interestingly, in an earlier study, Zorn *et al*. [132] demonstrated that in *Xenopus* embryos, the ectopic expression of XSOX17α, XSOX17β and XSOX3 ventralized embryos by inhibiting the Wnt pathway. They further showed that all three XSOX proteins bind to β-catenin via Armadillo repeats 3-6, that overlap with the TCF binding site (Armadillo repeats 4-9) on β-catenin [132]. Since plakoglobin is able to interact with SOX4, it may be possible that it also interacts with other SOX proteins, thereby inhibiting Wnt signaling. Finally, a recent report demonstrated that knockdown of desmoglein 3 in head and neck cancer cell lines resulted in increased nuclear plakoglobin levels and its interactions with TCF-4, that led to decreased TOPFLASH reporter activity and downregulation of β-catenin -TCF target genes (e.g. c-Myc, cyclin D1, MMP7; [133]). The inhibition of β-catenin-TCF-4 transcriptional activity by plakoglobin was also demonstrated in a recent study that reported the interaction of both catenins with the cellular prion protein PrP(c) [83, 134,135]. PrP(c) was shown to interact with actin, spectrin, annexin A2, plakoglobin, desmoglein 2, desmoplakin and c-Src in epithelial cells. In polarized epithelial cells, PrP(c) showed primarily membranous/junctional distribution, whereas in actively proliferating cells it was localized to the nucleus [83]. This study also detected a pool of PrP(c) in lipid rafts in association with desmoglein 2 and c-Src. Subsequent studies by the same group showed PrP(c) participated in Hippo and Wnt signaling pathways. PrP(c) interacted not only with plakoglobin but also with β-catenin and TCF-4 in both the cytoplasm and nucleus. Futhermore, the interaction of PrP(c) with β-catenin-TCF-4 stimulated its transactivation, whereas PrP(c)-plakoglobin interactions decreased it [134, 135].

### iii. Intracellular interactions

Plakoglobin may act as a tumor and metastasis suppressor through its interactions with various intracellular partners, thereby altering their levels, localization or function. In support of this scenario, previous work using *Xenopus* embryos showed that plakoglobin protein sequestered exogenously-expressed TCF proteins in the cytoplasm, resulting in a decrease in TOPFLASH reporter activity [41].

We recently showed that plakoglobin interacted with nucleophosmin (NPM; [56]), a nucleolar phosphoprotein whose role in tumorigenesis is largely dependent on its subcellular distribution [136, 137]. We showed that plakoglobin expression in MDA-MB-231 cells resulted in increased NPM protein levels and its redistribution from the cytoplasm and nucleoplasm, where it is thought to function as an oncogene, into the nucleolus, where it is typically localized in untransformed cells [56, 136–138]. Therefore, plakoglobin, through its interactions with NPM, altered NPM protein levels and localization concurrent with the decreased growth, invasive and migratory properties of MDA-MB-231-PG cells [56].

In addition, we previously showed that plakoglobin expression in SCC9 and MDA-MB-231 cells resulted in increased protein levels of the metastasis suppressors Nm23-H1 and -H2 [53, 58]. Following expression, plakoglobin interacted with both Nm23-H1 and -H2 leading to a change in the distribution of a significant proportion of Nm23 from cytoplasmic to membrane-associated. Our results suggested that plakoglobin increased the levels of Nm23 proteins via its interactions with Nm23, which potentially led to increased protein stability. A number of subsequent studies showed a critical role for Nm23 in cell-cell adhesion. Nm23 knockdown in hepatoma and colon carcinoma cells led to adherens junction dissociation, β-catenin nuclear translocation, β-catenin-TCF transactivation and upregulation of MMPs, concurrent with increased cell motility and migration [139]. Nm23 was shown to play critical roles in regulating permeability and barrier function of endothelial monolayers [140] via interacting with plakoglobin and EMMPRIN in the stabilization of endothelial junctions [141]. This study further suggested that EMMPRIN-plakoglobin interactions regulated actomyosin-dependent forces that were required for junction formation. Interestingly, plakoglobin also has been shown to interact with CPI-17, which inhibits MLC phosphatase [142]. Together, these studies point to a role for Nm23 and plakoglobin in migration inhibition and endothelial junction stability with implications for metastasis and angiogenesis.

### iv. Regulation of gene expression

Plakoglobin has been shown to be a repressor of the c-Myc (*MYC*) gene. In mouse keratinocytes, plakoglobin suppressed *MYC* expression in a LEF-1 dependent manner, suggesting that when plakoglobin interacted with LEF-1, this complex was unable to promote gene expression [143]. Furthermore, the plakoglobin-mediated suppression of *MYC* was similar in both wild type and β-catenin-null keratinocytes, demonstrating that plakoglobin could regulate gene expression independent of β-catenin. Finally, chromatin immunoprecipitation experiments with plakoglobin antibodies demonstrated that plakoglobin and LEF-1 associated with the *MYC* promoter in keratinocytes undergoing growth arrest, suggesting that the downregulation of c-Myc gene expression was a possible reason for the suppression of cell growth by plakoglobin [143].

Further evidence of a role for plakoglobin in the regulation of gene expression came from a recent study, which demonstrated that plakoglobin regulated the expression of the desmosomal cadherin desmocollin-2 in keratinocytes through interactions with LEF-1 [144]. The plakoglobin-mediated activation of the desmocollin-2 gene (*DSC2*) promoter was dependent on a functional LEF-1 binding site.

To identify potential plakoglobin target genes, proteins and mechanisms by which plakoglobin may regulate gene expression and in turn, tumorigenesis and metastasis, we performed proteomic and microarray experiments using plakoglobin-null cell lines and their plakoglobin-expressing transfectants. Various transfectants with different levels of expression and subcellular localization of plakoglobin were developed to specifically assess its role at the membrane, in the cytoplasm and in the nucleus [53, 72, 145]. Detailed analyses of these studies have suggested that plakoglobin may regulate gene expression by multiple mechanisms, either in the cytoplasm, through the sequestration of factors involved in regulating gene expression, or in the nucleus, by promoting and/or repressing gene expression itself (Figure 4; also see [18] for details). In the microarray experiments, we identified several p53 target genes that were differentially expressed following plakoglobin expression in various cell lines, suggesting that plakoglobin and p53 may coordinately regulate gene expression. Subsequently, we showed that plakoglobin interacted with p53 both in the cytoplasm and the nucleus, and this interaction was mediated by the DNA-binding domain of p53 and C-terminal transactivation domain of plakoglobin [57, 146]. Using transfectants expressing wild type and various fragments and deletions of p53 and plakoglobin, we demonstrated that wild type p53 and plakoglobin cooperated to decrease cell growth and acted synergistically to reduce migration and invasion [146]. We also have shown that together, p53 and plakoglobin regulated the expression of a number of p53 target genes, including the tumor suppressors 14-3-3σ and Nm23-H1, and the tumor promoter SATB1 [57, 58]. Using subcellular fractionation in conjunction with coimmunoprecipitation experiments, we showed that plakoglobin interacted with both wild type and mutant p53. Chromatin immunoprecipitation experiments showed that the two proteins also associated with the promoter of the *SFN* gene (which encodes 14-3-3σ). In addition, we showed that mutant p53 protein only associated with its target gene promoters in the presence of plakoglobin. Furthermore, using luciferase reporter assays, we showed that the transcriptional activity of both wild type and mutant p53 proteins was enhanced in the presence of plakoglobin [57]. Consistent with our observations, it was previously shown that plakoglobin promotes the expression of the tumor suppressor PML, a known p53 target gene [147]. More recently, Sechler *et al*. [148] showed that plakoglobin induced the expression of hepatocyte growth factor activator inhibitor Type I (HAI-1), an upstream inhibitor of c-met, in a p53-dependent manner, to reduce migration in NSCLC cells [148].

We have observed that plakoglobin interacts with a number of different mutant p53 proteins in various cell lines (e.g. SCC9, A431, MDA-231, ES-2; [57, 58, 146, 149]). Furthermore, plakoglobin appeared to help mutant p53 associate with its target gene promoters- an association that is absent when plakoglobin is not expressed. As such, our data suggests that plakoglobin may help promote the wild type tumor suppressor activities of mutant p53 proteins that have otherwise lost these activities, with the end result of a decreased transformed cell phenotype. This is extremely important when considering that approximately 50% of all tumors express a mutant p53 protein [150–153].

**Figure 5:**
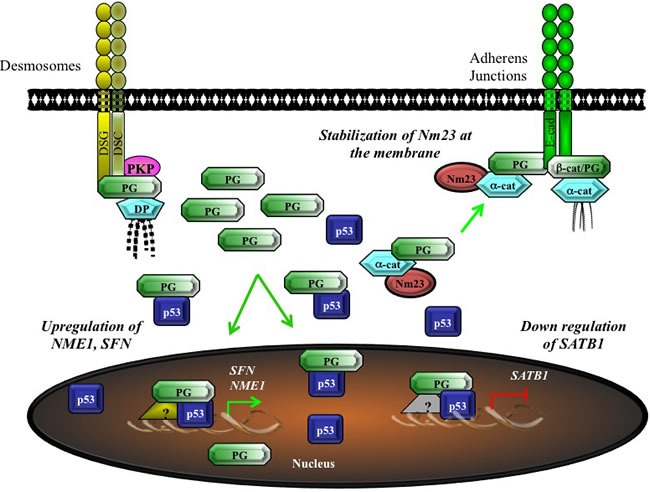
Potential model for the tumor/metastasis suppressor activity of plakoglobin via the regulation of gene expression Cytoplasmic, non-junctional plakoglobin may regulate tumorigenesis and metastasis by multiple mechanisms. Plakoglobin interacts with the metastasis suppressor Nm23 and increases its protein levels and localization at the membrane. Plakoglobin also interacts with the transcription factor p53 and promotes the expression of tumor and metastasis suppressor genes (e.g. *NME1, SFN)* and suppresses the expression of oncogenes (e.g. *SATB1)*. α-cat, α-catenin; β-cat, β-catenin; DP, desmoplakin; Nm23, nonmetastatic protein 23; PG, plakoglobin; SFN, stratifin.

We have shown that plakoglobin and p53 associated with and repressed expression from the *SATB1* promoter, which encodes SATB1 protein, the oncogenic chromatin remodeling factor [58; 154]. We have also shown that a number of SATB1 target genes were differentially expressed following plakoglobin knockdown in MCF-7 cells [58]. Furthermore, using chromatin immunoprecipitation experiments and luciferase reporter assays we have found that plakoglobin and p53 interacted with and promoted expression from the *NME1* promoter, which encodes the metastasis suppressor Nm23-H1 [58]. Our findings are consistent with a previous report that showed Nm23-H1 mRNA levels were decreased following plakoglobin knockdown in breast cancer cells [54]. We further showed that breast, squamous cell, ovarian and non-small cell lung carcinomas that expressed plakoglobin were less proliferative, migratory and invasive compared to those that did not express plakoglobin [29, 58, 146, 149]. These results are in agreement with other studies that have demonstrated that plakoglobin expressing cells are considerably less proliferative and migratory than their non-plakoglobin expressing counterparts [30, 44–52, 54, 55, 155–157]. More importantly, our data also suggests that the plakoglobin-p53 complexes are both activating (in the case of the *SFN* and *NME1* genes) and repressive (*SATB1* gene). A summary of our results is shown in Figure 5.

Finally, as mentioned earlier, Todorovic *et al*. [51] previously showed that plakoglobin regulates cell motility by regulating fibronectin and Rho-dependent Src signaling. In this study, plakoglobin expression also resulted in increased levels of fibronectin mRNA due to its increased stability, as determined by the use of the transcription inhibitor Actinomycin D [51]. This finding suggests that in addition to its role in regulating gene expression at the level of transcription, plakoglobin may also regulate gene expression post-transcriptionally. However, how plakoglobin does so remains unclear.

## CONCLUDING REMARKS

Following its initial discovery and early characterization, plakoglobin was primarily regarded as a protein whose function was limited to maintaining proper cell-cell adhesion. However, recent findings have clearly pointed to a more active role for plakoglobin in the regulation of tumorigenesis and metastasis through multiple mechanisms, including the regulation of gene expression. While characterizing the signaling function of plakoglobin in the absence of β-catenin has been a complicated endeavor, there is now ample evidence supporting the role of plakoglobin as an important player in tumorigenesis and metastasis. Plakoglobin interaction with p53 and its apparent ability to restore the wild type transcriptional activity of mutant p53 proteins may have significant therapeutic implications. The identification of the domains in plakoglobin that mediate its interactions with p53 can be used to design small therapeutic peptides/drugs that mimic these interactions and reactivate the wild type activity of mutant p53. These peptides/drugs may potentially have high, specific potency but avoid the low efficacy and toxicity of drugs that have not been derived from a naturally occurring cellular protein. This is particularly important since 50% of all tumors and >80% of metastatic tumors have non-functional p53 and the reactivation of p53 is currently being actively explored as a potentially effective therapeutic intervention in the treatment of various cancers.

## Abbreviations

AML: Acute myeloid leukemia
APC: Adenomatous polyposis coli protein
CML: Chronic myeloid leukemia
CTC: Circulating tumor cell
DNMT: DNA methyltransferase
EMT: Epithelial to mesenchymal phenotypic transition
ESC: Embryonic stem cell
HAI: 1-Hepatocyte growth factor activator inhibitor Type I
HDAC: Histone deacetylase
hGH: Human growth hormone
HSC: hematopoietic stem cell
HSPC: hematopoietic stem progenitor cell
JUP: Junction plakoglobin
LEF: Lymphoid enhancer factor
M: CSFR-Macrophage colony-stimulating factor receptor
MET: Mesenchymal to epithelial phenotypic transition
Nm23: Nonmetastatic protein 23
NPM: Nucleophosmin
NSCLC: Non-small cell lung carcinoma
PrP(c): Cellular prion protein
SFN: Stratifin (14-3-3σ)
TCF: T-cell factor
TOPFLASH-TCF: luciferase reporter vector
